# The Effect of Informational Podcasts on Shared Decision-Making, Anxiety, and Patient Satisfaction in Hospital Visits: Intervention Study

**DOI:** 10.2196/81485

**Published:** 2026-04-29

**Authors:** Jannie Christina Frølund, Anders Løkke, Hanne Irene Jensen, Flemming Skjøth, Ingeborg Farver-Vestergaard

**Affiliations:** 1 Department of Medicine, Lillebaelt Hospital, Beriderbakken 4, Vejle, 7100, Denmark, +45 79406949; 2 Department of Regional Health Research, University of Southern Denmark, Odense, Denmark; 3 Department of Anaesthesiology and Intensive Care, Lillebaelt Hospital, Vejle and Kolding, Denmark; 4 Research Support Unit, Lillebaelt Hospital, Vejle, Denmark

**Keywords:** digital health, podcasts, patient education as topic, shared decision making, health literacy, patient engagement, anxiety, patient satisfaction, eHealth communication

## Abstract

**Background:**

Podcasts provide a platform for delivering patient information. They have the potential to enhance patient engagement in shared decision-making (SDM), reduce anxiety in relation to hospital visits, and improve patient satisfaction. However, their impact on these outcomes in the context of hospital visits remains underexplored.

**Objective:**

This study aimed to examine whether podcasts influence patients’ (1) engagement in SDM, (2) anxiety after the hospital visit, and (3) satisfaction with the hospital visit.

**Methods:**

A quasi-experimental design with a nonequivalent comparison group was used. The study was conducted in 3 specialized outpatient clinics at a Danish hospital. Patients were allocated to one of 2 groups: the intervention group, which received access to informational podcasts in addition to standard written information before their hospital visit, and the comparison group, which received only the standard written information. All patients received validated questionnaires to assess SDM (9-item Shared Decision-Making Questionnaire [SDM-Q-9]), anxiety (State-Trait Anxiety Inventory-State), and satisfaction after the consultation.

**Results:**

A total of 240 patients participated. Compared with the control group, the intervention group showed a 15% higher level of SDM (SDM-Q-9) scores (adjusted relative difference=1.15, 95% CI 1.05‐1.26; *P*=.18). Subgroup analyses indicated a statistically significant effect among patients with low health literacy (adjusted relative difference=1.81, 95% CI 1.42‐2.32; *P*=.002). Anxiety scores were 9% lower (adjusted relative difference=0.91, 95% CI 0.84‐0.99; *P*=.23), and satisfaction with previsit information increased by 14% (adjusted relative difference=1.14, 95% CI 1.07‐1.21; *P*=.003).

**Conclusions:**

Informational podcasts, provided as a supplement to traditional written information, may offer modest support for patient engagement in SDM, particularly among patients with low health literacy. Podcasts also appear to improve satisfaction with previsit information more broadly. Effects on previsit anxiety were inconclusive.

## Introduction

Effective communication and information exchange between patients and health care providers is essential for optimal treatment outcomes. It plays an important role in shared decision-making (SDM), patient satisfaction, and reduces anxiety related to health consultations [1-4].

Podcasts provide a platform for delivering patient information and supporting preparation for hospital visits [5]. Qualitative research suggests that podcasts can alleviate patients’ anxiety related to hospital visits, foster a sense of trust in health care professionals, and enhance their understanding of empowerment [6]. Despite the rising popularity of podcasts in general society, there is a lack of research investigating their impact on SDM, anxiety, and patient satisfaction with hospital visits [5,6].

SDM is a collaborative process that fosters patient engagement and empowerment by actively involving patients in decisions about their care [7]. This approach, which prioritizes patient preferences, has been shown to improve treatment adherence, satisfaction, quality of life, and health outcomes by integrating patients’ perspectives into the decision-making process [8-12]. SDM is particularly relevant in clinical contexts characterized by diagnostic uncertainty and complex treatment considerations. In patients referred with suspected lung cancer, consultations often involve discussions of further diagnostic testing and potential malignancy, which may be associated with substantial psychological distress [13]. Patients with chronic obstructive pulmonary disease (COPD) frequently face decisions related to symptom management and inhaler choice; SDM has been shown to facilitate inhaler choice and support adherence and satisfaction in patients with newly-diagnosed COPD [14]. SDM interventions in chronic respiratory conditions have also been associated with improved medication adherence [15]. Similarly, patients referred for evaluation of sleep apnea are introduced to diagnostic procedures and possible treatment options, such as continuous positive airway pressure, which require long-term engagement and adherence [16]. In these contexts—often representing patients’ first hospital visit for assessment and possible initiation of treatment—adequate preparation may be essential for meaningful participation in SDM. Beyond its clinical implications, SDM is closely linked to patients’ psychological responses to hospital visits and their evaluation of care. Meaningful participation in decision-making presupposes that patients feel adequately informed and prepared. When patients understand the purpose of the visit and the potential diagnostic or treatment decisions to be addressed, they are more likely to engage actively in SDM. Such preparedness may simultaneously reduce uncertainty-related anxiety and contribute to greater satisfaction with the hospital visit. Conversely, insufficient preparation may limit participation, increase psychological distress, and negatively influence satisfaction [17]. Engagement in SDM, anxiety, and patient satisfaction can therefore be understood as interrelated dimensions of the hospital visit experience rather than independent outcomes. With the increasingly widespread use of smartphones and internet access, podcasts can be used to deliver information to a broad audience, enabling patients to access information and prepare for the hospital visit at their convenience. Increasingly, podcasts are being used as a supplement to traditional written patient information to support patient preparation for hospital visits, particularly before an initial consultation focused on diagnostic clarification and potential treatment planning [5,6]. Qualitative studies suggest that podcasts can foster a sense of trust, reduce anxiety, and enhance patients’ confidence and engagement during hospital visits [6]. Despite their potential benefits, the impact of podcasts on SDM remains underexplored, as they are relatively new in the context of patient information [5,6].

Informed patients are less susceptible to anxiety and stress surrounding hospital visits and medical procedures [2]. When faced with health-related concerns, patients often seek information as a means of alleviating their anxiety and gaining a better understanding of their medical conditions and treatment options [18]. Written patient information—such as leaflets—can be effective in reducing anxiety, particularly when well-designed and appropriately used [19-22]. However, there are significant challenges related to traditional patient information [23]. In addition, patients with limited literacy, cognitive impairments, or disabilities may face barriers to accessing and comprehending written materials, further contributing to health inequalities [24-27]. Medical jargon and a lack of engagement can also limit the effectiveness of written communication [28-30]. This highlights the need to explore alternative formats—such as podcasts—that, through the use of voice, may provide more accessible, relatable, and emotionally supportive ways of informing and preparing patients [6].

Although the literature on the efficacy of podcasts as patient information is growing, more research is needed on their effect in the context of hospital visits. The objective of this study was to address this gap by investigating the effect of informational podcasts on patients’ (1) engagement in SDM, (2) anxiety following the hospital visit, and (3) satisfaction with the hospital visit.

## Methods

### Design

This study used a quasi-experimental design with a nonrandomized comparison group to evaluate the effect of podcasts on patients’ engagement in SDM, anxiety related to the hospital visit, and overall satisfaction.

In this natural experimental setup, patients recruited before the implementation of podcasts served as the comparison group, while those enrolled afterwards constituted the intervention group. Randomization was not feasible in the clinical context. As highlighted by the Medical Research Council [31], natural experiments can provide valid evidence of intervention effects when randomization is impractical—provided that contextual factors, assumptions, and potential biases are systematically addressed.

### Settings and Participants

The study was conducted in 3 specialized outpatient clinics at a Danish hospital—lung cancer diagnostics, COPD, and sleep apnea clinics. Patient recruitment for the comparison group occurred from 2021 to 2022, before the podcasts were implemented. The podcasts were implemented at different times in each clinic (April 2022 [lung cancer], January 2023 [sleep apnea], and March 2023 [COPD]). Patients in the intervention group were enrolled roughly 3 months after podcast implementation to ensure the podcasts were integrated into standard previsit information.

Patients met the following inclusion criteria:

- Newly referred for suspected lung cancer, COPD, or sleep apnea
- Aged 18 years or older
- Able to speak and understand Danish

Patients with severe mental illness, cognitive impairments, or terminal conditions were excluded to ensure meaningful participation and reduce confounding from communication or health status challenges. Both comparison and intervention groups consisted of 40 patients per clinic. Recruitment took place on selected days, aligned with the clinic’s capacity and available resources.

### Intervention

We implemented 3 user-centered podcasts [5], each tailored to assist either patients with suspected lung cancer [32], COPD [33], or sleep apnea [34]. These podcasts provide information about why patients were referred to the clinic, what to expect during their visit, and how to prepare. An overview of the shared structure and core components of the podcasts is presented in Table 1. The development of these podcasts was guided by the “empathy map” method [5], which helped identify patients’ concerns and informational needs before their hospital visits. This method ensured the podcasts were tailored to each patient group’s unique needs [35-37]. Adequate preparation before hospital visits is theorized to simultaneously facilitate engagement in SDM, reduce uncertainty-related anxiety, and enhance overall satisfaction with the hospital visit. Patients who feel informed and confident in their understanding of the hospital visit are more likely to participate meaningfully in decision-making and evaluate the experience positively, whereas insufficient preparation may limit engagement, increase psychological distress, and reduce satisfaction [17]. The podcasts were therefore intended not only to provide information but to influence these interrelated outcomes.

**Table 1.** Overview of condition-specific podcasts^a^.

Section	Content	Purpose
Target audience and considerations	Patients referred to outpatient clinics (lung cancer, COPD^b^, or sleep apnea). Common patient concerns include anxiety, uncertainty, and a need for clear guidance.	Tailor content to patient needs; acknowledge emotions; build trust.
Introduction	Brief host introduction and explanation of podcast purpose.	Orient patients; set expectations.
Patient feelings	Acknowledge common emotional responses (eg, nervousness, worry, and frustration).	Reduce anxiety; show empathy.
Core topics	Reasons for referral, what to expect during the hospital visit, stepwise outline of the visit or diagnostic process, practical advice while waiting for results, and summary and key take-home messages	Provide structured, relevant information; improve comprehension and readiness for hospital visit.
Practical advice	Encourage patients to ask questions, prepare notes, talk with relatives, and maintain daily routines.	Support coping; reduce anxiety; empower patients.
Summary and reinforcement	Recap main points covered in the podcast.	Reinforce key messages; support retention and engagement.

a Podcasts were developed using the empathy map method [35-37] to ensure content aligns with patient concerns and informational needs. Each podcast follows the same general structure while addressing condition-specific aspects relevant to the respective patient group.

b COPD: chronic obstructive pulmonary disease.

During the study period, the clinics experienced no significant organizational changes, ensuring a stable environment for assessing the intervention and maintaining consistent care quality.

### Patient Groups

For each condition (suspected lung cancer, COPD, and sleep apnea), 2 patient groups were included—a comparison group seen before the implementation of the podcasts, and an intervention group seen after. All patients received standard written information about their upcoming consultation via the national digital post system 2 to 30 days before their scheduled visit, depending on clinic procedures. In the intervention group, this letter also included a QR code and link to the condition-specific podcast, encouraging patients to listen before attending their appointment. Patients in the comparison group did not have access to the podcasts.

### Data Collection

All patients received a questionnaire on the same day or the day after their consultation to assess their experiences and perceptions regarding the hospital visit. Patients had the option to complete the questionnaire in either written or electronic format. Those who selected the paper version received specific instructions based on their condition; patients with suspected lung cancer or sleep apnea were asked to return the completed questionnaire during their scheduled visit the next day, while patients with COPD were required to complete it immediately due to the infrequency of their hospital appointments. This ensured that all paper responses were collected. For patients opting for the electronic version, the questionnaire was sent the day after the consultation, with follow-up reminders issued after 2 and 4 weeks. No further action was taken if the questionnaire remained unreturned after this period. For patients who received the paper version, no additional reminders were sent beyond the initial instructions given at the hospital.

### Outcome Measures

This study assessed key outcomes using validated instruments to evaluate the impact of podcasts on engagement in SDM, preconsultation anxiety levels, and overall satisfaction. The primary outcome was:

- SDM: The 9-item Shared Decision-Making Questionnaire (SDM-Q-9) consists of 9 statements rated on a 6-point scale (0‐5) from “completely disagree” (0) to “completely agree” [5,38]. Total scores range from 0 to 45, with higher scores indicating a greater perceived SDM. The SDM-Q-9 measures concrete behaviors and perceptions, such as whether patients felt informed, listened to, and involved in choices about their care. In this study, the validated Danish version of the SDM-Q-9 was used [39], which has been shown to retain the psychometric properties of the original instrument.

Secondary outcomes included:

- Anxiety: State anxiety was assessed using the State-Trait Anxiety Inventory-State (STAI-S) questionnaire, a validated 20-item self-report measure designed to evaluate transient anxiety levels in response to a specific situation [40]. The STAI-S measures transient, situation-specific feelings of anxiety, such as nervousness, worry, or tension, related to the hospital visit. Each item is rated on a 4-point Likert scale, ranging from 1 (not at all) to 4 (very much), with a total score ranging from 20 to 80. Higher scores indicate greater levels of state anxiety.
- Patient satisfaction was measured using four validated questions from the National Danish Survey of Patient Experiences (Landsdækkende Undersøgelse af Patientoplevelser [LUP]). The LUP questionnaire assesses patients’ experiences with the Danish health care system. The questions addressed key aspects of the consultation process—satisfaction with preconsultation information, comfort during the consultation, adequate responses to their questions, and overall satisfaction. Participants responded using a 5-point Likert scale, ranging from “very dissatisfied” (1) to “very satisfied” (5) [41].

In addition, this study examined how various factors, including patient characteristics, provider attributes, and consultation characteristics, influenced the effect of podcasts. These factors were as follows:

- Sociodemographic information: Age, gender, social status, education level, and work status. Some response categories within variables (eg, education level and work status) were collapsed due to limited responses.
- Health literacy: Patients’ health literacy was measured using the 16-item European Health Literacy Survey [42]. The questionnaire captures self-reported difficulties in accessing, understanding, appraising, and applying health-related information concerning health care, disease prevention, and health promotion [42-44]. Each item is rated on a 4-point Likert scale from “very easy” to “very difficult.” Responses were dichotomized, with “very easy” and “easy” scored as 1, and “difficult” and “very difficult” scored as 0. The resulting total score ranged from 0 to 16 and was categorized into 3 levels: inadequate (0‐8), problematic (9-12), and adequate (13-16).
- Relative presence: Recorded whether relatives were present during the consultation.
- Consultation duration: The duration of the consultation was documented.
- Podcast exposure: Self-reported listening to the condition-specific podcast was assessed in the follow-up questionnaire for patients in the intervention group.

### Ethical Considerations

The Regional Committees on Health Research Ethics for Southern Denmark reviewed the study protocol and determined that formal ethical approval was not required (20202000‐222). The study was registered with the Regional Danish Data Protection Agency (20/55212).

All patients gave informed consent before completing the questionnaires. Participation was voluntary, and confidentiality was ensured. Departmental staff provided patients with oral and written information about the study. Signed consent forms were collected, and patients were informed that they could withdraw without affecting their treatment or care.

### Statistical Analyses

Descriptive statistics were used to summarize patient characteristics and outcomes. Between-group differences were summarized by standardized differences using Cohen *d*. The relative effect of the intervention with 95% CIs was further assessed for significance assuming independence between subjects using a log-normal linear model. The intervention effects were reported as crude and adjusted estimates. Adjusted estimates accounted for the following predefined potential confounders: sex, age, clinic, educational level, work status, health literacy, presence of relatives during the consultation, and consultation duration. All analyses were performed using STATA 18 (StataCorp LLC) with a significance level set at *P*<.05.

### Sample Size Estimation

A power calculation based on the SDM-Q-9 indicated that 210 patients, evenly distributed between the intervention and comparison groups, would be needed to detect a clinically relevant difference in patient outcomes with a medium effect size (Cohen *d*=0.50), 95% power, and a 2-sided α of .05. To account for a 10% dropout rate, we aimed to recruit 232 patients.

## Results

### Overview

**Figure 1.**
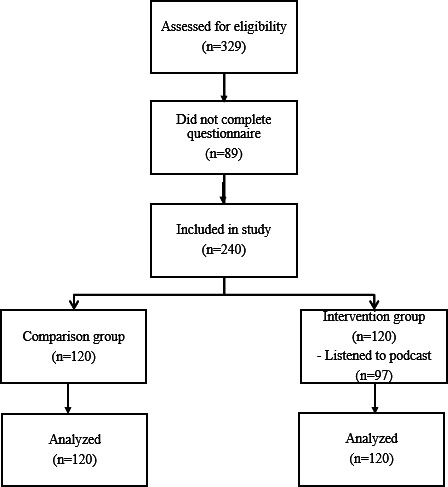
Flow of patients through the study, including podcast exposure and questionnaire completion.

A total of 240 patients were included in the study, with 120 allocated to the comparison and intervention groups, respectively. An additional 89 patients initially consented but did not complete the questionnaires. Figure 1 illustrates the flow of patients through the study, including the number of patients who actually listened to the podcast in the intervention group.

Overall, the groups were comparable across most demographic and clinical variables, including age, disease distribution, and employment status. Slight group differences were observed in education level and health literacy. The comparison group had a higher proportion of patients with a university education (63/120, 52% vs 49/120, 41%), whereas more patients in the intervention group reported only primary schooling (30/120, 25% vs 12/120, 10%). Health literacy scores were slightly higher in the intervention group (mean 13.0, SD 4.1, range 0-16 vs mean 12.5, SD 3.6, range 0-16), and a greater share of patients in this group fell into the “high literacy” category (91/120, 75% vs 73/120, 60%).

The consultation time was similar between groups (mean 34, SD 25.3, range 5-180 min), as was the proportion of patients who attended with a relative (54/120, 45% in the intervention group and 53/120, 44.2% in the comparison group). Patients in the intervention group were sent a QR code to access the podcast before their hospital visit, and 81% (n=97) of these patients reported having listened to the podcast. Patients in the comparison group did not have access to the podcast. Characteristics of the included patients can be found in Table 2.

**Table 2.** Patient characteristics (N=240).

Characteristics	All (N=240)	Comparison group (n=120)	Intervention group (n=120)
Sex (female), n (%)	103 (43)	59 (49)	44 (37)
Age, mean (SD; range)	61.7 (12.2; 26-99)	60.9 (12.9; 26-99)	62.4 (11.4; 38-86)
Disease, n (%)			
Suspected lung cancer	80 (33)	40 (33)	40 (33)
Suspected COPD^a^ or COPD	80 (33)	40 (33)	40 (33)
Suspected sleep apnea	80 (33)	40 (33)	40 (33)
Relationship status (1 patient in the intervention group did not respond), n (%)			
Single	72 (30)	31 (26)	40 (33)
Married or cohabiting	137 (70)	89 (74)	79 (66)
Educational level, n (%)			
Low (primary school)	42 (18)	12 (10)	30 (25)
Medium (high school or vocational education and training)	86 (36)	45 (38)	41 (35)
High (university)	112 (46)	63 (52)	49 (41)
Work status (1 missing in comparison group), n (%)			
Employed	92 (38)	47 (39)	45 (38)
Unemployed	23 (10)	11 (9)	12 (10)
Retired	99 (41)	50 (42)	49 (41)
Disability pension	25 (10)	11 (9)	14 (12)
Health literacy score, mean (SD; range)	12.9 (3.9; 0‐16)	12.5 (3.6; 0‐16)	13.0 (4.1; 0‐16)
Health literacy levels (HLS-EU-Q16^b^), n (%)			
Low	35 (14)	20 (17)	15 (13)
Medium	41 (17)	27 (23)	14 (12)
High	164 (68)	73 (60)	91 (75)
Relatives present at medical consultation, n (%)			
Yes	107 (45)	53 (44)	54 (44)
Duration of medical consultation, mean (SD; range)	34.1 (25.3; 5-180)	34.8 (26.6; 5-180)	33.4 (20.8; 5-150)
Have the patient heard the podcast			
Yes, n (%)	97 (40)	-	97 (81)
No, n (%)	143 (60)	120 (100)	23 (19)

a COPD: chronic obstructive pulmonary disease.

b HLS-EU-Q16: 16-item European Health Literacy Survey.

### Objective 1: Patients’ Engagement in SDM

Patients in the intervention group reported a mean SDM-Q-9 score of 31.5 (SD 0.98), compared with 27.4 (SD 1.02) in the comparison group. This difference was statistically significant in the unadjusted analysis but lost significance after adjusting for potential confounders (Table 3).

**Table 3.** Shared decision-making (SDM-Q-9^a^; score range 0‐45), anxiety (STAI-S^b^; score range 20-80), and patient satisfaction scores (range 1-5) in intervention (n=120) and comparison (n=120) groups (N=240).

Outcomes	Comparison group, mean (SD)	Intervention group, mean (SD)	Relative change (95% CI)^c^	Standardized effect size, Cohen *d* (95% CI)^d^	Crude *P* value^e^	Adjusted *P* value^f^
Primary outcome: shared decision-making, range: 0‐45						
Shared decision-making total score (SDM-Q-9)	27.4 (1.02)	31.5 (0.98)	1.15 (1.05‐1.26)	0.38 (0.12‐0.63)	.004	.18
Secondary outcome: anxiety, range: 20‐80						
Total Anxiety Score (STAI-S)	37.01 (12.14)	33.85 (10.66)	0.91 (0.84‐0.99)	0.28 (0.02‐0.53)	.03	.23
Secondary outcome: satisfaction, range: 1‐5						
Consultation comfort	4.14 (0.81)	4.38 (0.89)	1.06 (1.00‐1.11)	0.28 (0.03‐0.54)	.03	.17
Answers to questions	3.91 (1.04)	4.29 (0.88)	1.10 (1.04‐1.17)	0.41 (0.15‐0.66)	.002	.09
Previsit information	4.01 (1.15)	4.55 (0.88)	1.14 (1.07‐1.21)	0.53 (0.27‐0.79)	<.001	.003
Overall consultation satisfaction	3.91 (1.07)	4.33 (0.83)	1.11 (1.05‐1.18)	0.44 (0.19‐0.70)	<.001	.11

a SDM-Q-9: 9-item Shared Decision-Making Questionnaire.

b STAI-S: State-Trait Anxiety Inventory-State.

c The relative change (95% CI) indicates the proportional change between groups (comparison vs intervention) with a 95% CI.

d Standard effect size (Cohen *d*) is a standardized effect size that quantifies the difference between 2 groups in standard deviation units with a 95% CI.

e The crude *P* value represents the initial significance level without adjustments.

f The adjusted *P *value accounts for confounders (eg, gender, age, education, presence of accompanying relative, disease type, health literacy, and perception of health).

Subgroup analyses revealed consistently higher levels of SDM in the intervention group, compared with the comparison group. However, after adjustment, only patients with low health literacy in the intervention group showed statistically significant higher levels of SDM, relative to the comparison group. No significant differences were found across other demographic or clinical variables, including gender, age, education, employment status, relationship status, or the presence of a relative during the consultation (Table 4).

**Table 4.** Differences in shared decision-making scores (range 0‐45) across demographic and disease-related factors in intervention (n=120) and comparison (n=120) groups (N=240).

Characteristic	Comparison group, mean (SD)	Intervention group, mean (SD)	Relative effect (95% CI)^a^	Adjusted *P* value for interaction^b^
Sex				
Females	26.51 (1.47)	32.34 (1.59)	1.22 (1.05‐1.41)	—^c^
Males	28.25 (1.41)	31.01 (1.25)	1.10 (0.97‐1.24)	.83
Age (y)				
<67	26.56 (1.30)	29.99 (1.38)	1.22 (0.99‐1.29)	—
>67	28.78 (1.58)	33.62 (1.30)	1.17 (1.03‐1.33)	.29
Education				
Low (primary school)	30.58 (2.82)	34.27 (1.23)	1.12 (0.95‐1.32)	—
Medium (high school or vocational education and training)	25.93 (1.79)	30.85 (1.94)	1.19 (0.99‐1.43)	.83
High (university)	28.83 (1.35)	30.35 (1.59)	1.09 (0.95‐1.25)	.36
Work status				
Employed	28.32 (1.59)	33.45 (1.31)	1.10 (0.92‐1.31)	—
Unemployed	28.36 (2.71)	31.00 (2.91)	1.09 (0.84‐1.43)	.78
Retired	28.32 (1.59)	33.45 (1.31)	1.18 (1.03‐1.35)	.43
Disability pension	25.72 (4.11)	32.86 (1.84)	1.28 (0.96‐1.71)	.52
Relationship status				
Single	25.30 (1.93)	32.08 (1.36)	1.27 (1.08‐1.49)	—
Married or cohabiting	28.11 (1.90)	31.04 (1.32	1.10 (0.98‐1.24)	.28
Health literacy				
Low	17.55 (2.14)	31.80 (1.62)	1.81 (1.42‐2.32)	—
Medium	25.63 (1.84)	30.29 (3.06)	1.18 (0.94‐1.49)	.16
High	30.74 (1.21)	31.64 (1.18)	1.03 (0.92‐1.15)	.60
Suspected disease				
Suspected COPD^d^ or COPD	25.7 (1.62)	33.35 (0.82)	1.30 (1.15‐1.47)	—
Suspected lung cancer	31.38 (1.77)	34.00 (1.59)	1.08 (0.94‐1.25)	.11
Suspected sleep apnea	25.08 (1.75)	27.15 (2.21)	1.08 (0.88‐1.34)	.06
Relatives present				
Yes	25.79 (1.35)	28.64 (1.46)	1.11 (0.96‐1.28)	—
No	29.48 (1.56)	35.11 (1.06)	1.19 (1.06‐1.33)	.41

a Relative effect (95% CI): The relative effect (intervention × comparison), with a 95% CI.

b Adjusted *P* value for interaction: The adjusted *P *value for the interaction between variables and the effect (intervention × comparison).

c Not applicable.

d COPD: chronic obstructive pulmonary disease.

**Figure 2.**
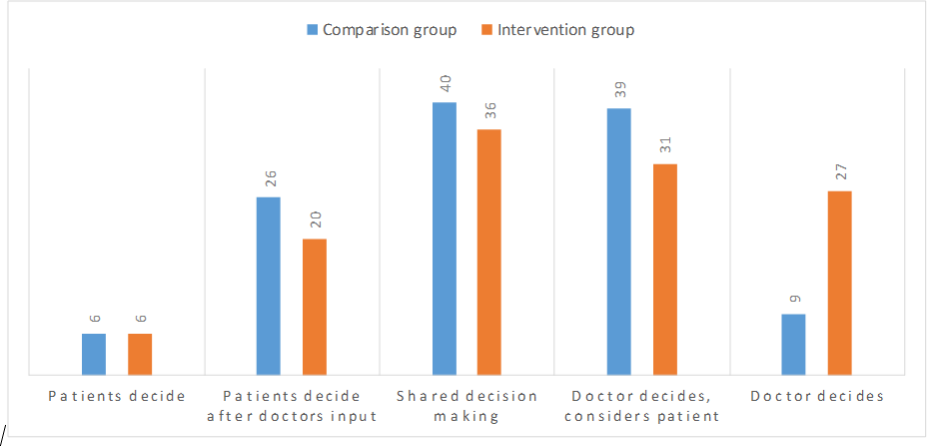
Preferences for involvement in shared decision-making based on the 9-item Shared Decision-Making Questionnaire in intervention (n=120) and comparison (n=120) groups (N=240).

Regarding preferences for decision-making roles, the majority of patients in both groups preferred SDM (40/120, 33% comparison vs 36/120, 30% intervention). Notably, more patients in the intervention group reported that the doctor decides alone (9/120, 8% vs 27/120, 23%, whereas fewer indicated that the doctor decides but considers the patient’s opinion (39/120, 33% vs 31/120, 26%; Figure 2).

### Objective 2: Patient Anxiety Associated With Hospital Visits

Patients in the intervention group reported lower anxiety levels than those in the comparison group (mean STAI-S 33.85, SD 10.66 vs 37.01, SD 12.14). However, this difference was no longer statistically significant after adjustment for potential confounders (Table 3).

Subgroup analyses showed lower anxiety scores in the intervention group across most demographic and clinical factors, with the largest differences seen in patients younger than 67 years, those with lower educational attainment, and those who were currently employed. However, none of the interaction effects reached statistical significance (Table 5). Similarly, no significant subgroup differences were observed for gender, relationship status, health literacy, suspected diagnosis (lung cancer, COPD, and sleep apnea), or presence of relatives during the consultation.

**Table 5.** Differences in anxiety levels (range 20‐80) across demographic and disease-related factors in intervention (n=120) and comparison (n=120) groups (N=240).

Characteristics	Comparison group, mean (SD)	Intervention group, mean (SD)	Relative chance (95% CI)^a^	Adjusted *P* value for interaction^b^
Sex				
Females	38.95 (1.67)	37.50 (1.83)	0.96 (0.85‐1.96)	—^c^
Males	35.14 (1.44)	31.73 (1.05)	0.90 (0.82‐1.00)	.72
Age (y)				
<67	38.59 (1.44)	33.04 (1.21)	0.86 (0.77‐0.95)	—
>67	34.37 (1.66)	34.97 (1.60)	1.02 (0.89‐1.16)	.85
Education				
Low (primary school)	40.54 (3.05)	33.64 (1.49)	0.83 (0.71‐0.97)	—
Medium (high school or Vocational Education and Training)	37.34 (2.01)	33.37 (1.67)	0.89 (0.77‐1.04)	.18
High (university)	36.10 (1.44)	34.36 (1.72)	0.95 (0.84‐1.08)	.06
Work status				
Employed	38.40 (1.76)	30.54 (1.29)	0.80 (0.70‐0.90)	—
Unemployed	42.84 (3.89)	38.81 (3.83)	0.91 (0.70‐1.18)	.73
Retired	34.24 (1.60)	35.30 (1.60)	1.03 (0.91‐1.17)	.22
Disability pension	36.21 (3.93)	35.14 (2.71)	0.97 (0.75‐1.25)	.38
Relationship status				
Single	38.42 (2.46)	34.24 (1.68)	0.89 (0.76‐1.04)	—
Married or cohabiting	36.52 (1.23)	33.54 (1.21)	0.92 (0.83‐1.01)	.18
Health literacy				
Low	43.81 (2.94)	37.56 (2.89)	0.86 (0.70‐1.06)	—
Medium	38.88 (2.24)	33.00 (2.37)	0.85 (0.69‐1.04)	.81
High	34.46 (1.33)	33.36 (1.13)	0.97 (0.88‐1.07)	.73
Suspected disease				
Suspected COPD^d^ or COPD	34.83 (1.64)	31.87 (1.32)	0.92 (0.81‐1.04)	—
Suspected lung cancer	39.99 (2.11)	37.47 (1.88)	0.94 (0.81‐1.08)	.96
Suspected sleep apnea	36.21 (1.93)	32.20 (1.69)	0.89 (0.77‐1.03)	.33
Relatives present				
Yes	38.49 (1.66)	35.77 (1.52)	0.93 (0.83‐1.05)	—
No	35.88 (1.66)	32.33 (1.24)	0.90 (0.81‐1.01)	.89

a Relative effect (95% CI): The relative effect (intervention × comparison), with a 95% CI.

b Adjusted *P *value for interaction: The adjusted *P *value for the interaction between variables and the effect (intervention × comparison).

c Not applicable.

d COPD: chronic obstructive pulmonary disease.

### Objective 3: Satisfaction With the Hospital Visit

The level of satisfaction with previsit information was significantly higher in the intervention group than in the comparison group (4.55 vs 4.01). This difference remained statistically significant after adjustment for potential confounders.

Differences between intervention and comparison groups in satisfaction levels across other domains did not remain statistically significant after adjustment (Table 3).

## Discussion

### Summary of Findings

To our knowledge, this is the first study to examine the impact of informational podcasts, as a supplement to standard written information, on SDM, consultation-related anxiety, and patient satisfaction among outpatients with suspected lung cancer, COPD, or sleep apnea.

The results suggest that podcasts appeared to provide modest support for patients’ engagement in SDM, especially among the subgroup of patients with low health literacy. Moreover, the implementation of podcasts was associated with higher satisfaction levels with preconsultation information. Effects on previsit anxiety were inconclusive, likely reflecting the multifactorial nature of anxiety and variations in patient engagement with digital content.

Taken together, these findings suggest that informational podcasts may serve as a modest, supplementary tool to traditional written materials, particularly for patients with low health literacy, rather than as a broadly transformative intervention across all patient groups.

### Objective 1: Patients’ Engagement in SDM

The findings of this study suggest that integrating podcasts into routine patient information may enhance patients’ engagement in SDM during outpatient consultations. Although the overall effect of the intervention was attenuated after adjustment for potential confounders, subgroup analyses revealed essential patterns. The most substantial improvements were observed among patients with low health literacy. These trends highlight the potential of podcasts to support patient populations who may otherwise face challenges engaging with written health information.

In Denmark, as in many other countries, a considerable proportion of the population experiences difficulties reading and comprehending health-related materials. National data indicate that up to 16% of adults struggle with basic reading and writing skills [24], which can undermine their ability to interpret written information, navigate health care systems, and participate meaningfully in SDM [43-45]. For such patients, audio-based formats like podcasts may provide a more accessible and effective alternative. Delivering complex patient information in an audio format that can be revisited at the patient’s pace promotes understanding in populations underserved by traditional written formats. While written materials also allow patients to revisit information, podcasts offer unique advantages, such as the ability to listen flexibly at home or on the go, which may better support patients who find it difficult to navigate or retain written content. This may explain why patients with low health literacy appeared to benefit more, as the podcast served as a more inclusive communication format. Notably, all 3 patient groups included in this study—those with suspected or diagnosed lung cancer, COPD, and sleep apnea—share a heightened risk of low health literacy [46-48]. These findings support that podcasts are not merely substitutes for written materials but represent a distinct and valuable communication format, particularly for patient groups with limited health literacy. The flexibility to listen at home or on the go and to revisit the content as needed may be especially advantageous for individuals who find it difficult to process complex written information or retain key messages during stressful health care encounters.

Nevertheless, while digital tools like podcasts increase access to patient information, they may also introduce new challenges. Older patients and patients with limited technological literacy may experience difficulties navigating digital platforms or downloading podcasts [49-51]. However, a qualitative study we conducted alongside this study found that podcasts were usable across age groups, suggesting that age was not a limiting factor for engagement [6].

These results are consistent with previous research indicating that patient-centered educational interventions can improve SDM, particularly among patients with limited health literacy [52].

It is important to note that not all patients seek the same degree of involvement. The findings of this study showed that a considerable proportion of the included patients preferred to defer decision-making to health care professionals. Notably, a larger proportion of patients in the intervention group than in the comparison group preferred that the health care professional decided without including the patient. The design of our study does not allow us to draw conclusions regarding causality, but this finding highlights the need for a nuanced, flexible approach to SDM—one that both encourages patient involvement and respects individual preferences regarding the extent of participation. This aligns with previous research suggesting that preference-sensitive SDM is most effective when patients are well-informed, supported, and empowered to define their role in the decision-making process [53,54].

### Objective 2: Patient Anxiety Associated With Hospital Visits

In this study, we did not find a clear effect of podcasts on patient anxiety. However, a previous qualitative interview study showed that many patients felt reassured and less anxious after listening to the podcasts. They explained that hearing health care professionals and fellow patients helped build trust and made them feel better prepared for their hospital visits [6].

There were indications that younger patients, those with lower educational attainment, and patients currently working might have experienced greater reductions in anxiety. These groups often face more stress due to competing responsibilities and may have lower health literacy, making them particularly vulnerable in health care settings. Although these trends were not statistically significant, they align with earlier research showing that patients from lower socioeconomic backgrounds tend to experience higher levels of hospital-related anxiety [55]. Podcasts may be particularly helpful for these groups by providing information in an accessible and easy-to-understand format, but further studies are needed to confirm this.

At the same time, anxiety around hospital visits is influenced by many factors beyond just information. Fear of diagnosis, past experiences, and the stressful nature of hospital visits contribute [56]. Information alone is unlikely to completely remove anxiety. It is also important to acknowledge that some anxiety is natural and appropriate when facing potentially serious health issues [57]. Attempting to eliminate anxiety entirely may neither be realistic nor helpful.

Overall, these findings suggest that podcasts can support patient preparation and potentially alleviate some anxiety; however, they should be part of a broader approach that includes empathetic communication, emotional support, and continuity of care to address patients’ emotional needs fully.

### Objective 3: Satisfaction With the Hospital Visit

This study found that integrating podcasts into preconsultation preparation was associated with improved patient satisfaction, particularly in terms of satisfaction with the previsit information. This suggests that podcasts can meaningfully enhance patients’ perception of the quality, clarity, and relevance of the information provided before hospital visits. These findings support previous research that demonstrates the positive impact of podcasts on patient satisfaction in various health care settings. In a qualitative study exploring patient experiences with podcasts, patients reported feeling more informed, prepared, and empowered during hospital visits [6].

Information provided before hospital visits shapes patients’ expectations and readiness for health care discussions. Effective communication is central to patient-centered care, enabling individuals to understand their diagnoses and treatment options and to participate actively in decision-making processes [1,58]. When patients are inadequately prepared, they may experience confusion, unmet expectations, and dissatisfaction with their care experience [59,60]. The podcasts evaluated in this study likely improved satisfaction by presenting condition-specific information in a format that was both accessible and engaging, helping patients to feel more confident and informed before their appointments.

Podcasts may add value by enabling repeated listening, allowing for more personalized preparation, and offering a human and conversational tone that differs from traditional written materials [5,6].

### Integration of SDM, Anxiety, and Patient Satisfaction

Engagement in SDM, patient anxiety, and satisfaction with the hospital visit are closely interrelated dimensions of the patient experience. Meaningful participation in SDM presupposes that patients feel adequately informed and prepared; when patients understand the purpose of the visit and the potential diagnostic or treatment decisions to be addressed, they are more likely to engage actively in decision-making, which may simultaneously reduce uncertainty-related anxiety and enhance satisfaction [17]. However, the effects of information are not uniform. For some patients, additional information—such as podcasts detailing diagnostic procedures or potential consequences of illness—may reduce anxiety by improving preparedness. For others, exposure to detailed information may temporarily increase state anxiety until coping strategies are established, which in turn can hinder engagement in SDM [61,62]. This dynamic is particularly relevant for patients referred for evaluation of suspected lung cancer, COPD, or sleep apnea, all of whom face complex clinical decisions requiring understanding and adherence to recommended procedures [14-17]. Informational podcasts, provided as a supplement to traditional written information, may modestly support engagement in SDM, improve understanding of pre-visit procedures, reduce uncertainty-related anxiety for some patients, and enhance satisfaction with the hospital visit. The effects observed at the group level were limited, highlighting that podcasts function primarily as a supplementary tool rather than a standalone intervention, and suggesting that their benefit may be most pronounced in specific patient subgroups, such as those with lower health literacy [14-17,61,62].

### Implications for Practice

The findings of this study have important implications for clinical practice, particularly in the context of supporting SDM. While podcasts can be a valuable supplement to standard written patient information, they should not be viewed as a standalone solution. Instead, they represent one of several elements that can help prepare patients for more active involvement in health care decisions.

For successful implementation, it is essential to recognize that patients have diverse needs and varying levels of health literacy. Particularly for individuals with low health literacy, additional support may be necessary to ensure they comprehend the information and feel confident in making informed choices.

When used appropriately, podcasts can help patients feel more informed and better prepared for hospital visits. However, their effectiveness depends on integration with broader communication strategies and a continuous focus on personalized support. In this way, podcasts can make a meaningful contribution to a more patient-centered approach to care.

### Strengths and Limitations

This study offers several notable strengths. First, its intervention design, including an intervention and comparison group, allows for a direct comparison of patient outcomes before and after the podcast implementation. This design strengthens the ability to attribute observed changes to the intervention.

The podcasts were developed using the “empathy map” methodology, ensuring the content was tailored to address the specific concerns and informational needs of patients with lung cancer, COPD, and sleep apnea. This user-centered approach enhances the relevance and potential effectiveness of the intervention.

The study implemented a robust evaluation framework, incorporating multiple validated outcome measures, including questionnaires that assessed SDM, patient satisfaction, and anxiety levels using the State-Trait Anxiety Inventory. This comprehensive evaluation provides a thorough understanding of the intervention’s impact. Furthermore, the sample size of 240 patients, evenly distributed across three clinical conditions and both intervention and comparison groups, improves statistical power for the primary outcome and the generalizability of the findings. This diverse sample provides valuable insights into how podcasts can be adapted to meet the needs of different patient groups.

Despite its strengths, the study has several limitations. Although the controlled design allows for comparison between groups, other factors, such as variation in disease severity and differences in provider interactions, may still have influenced outcomes. The study was conducted at a single site in Denmark, which may limit generalizability to other health care settings or populations with different cultural contexts and health care systems. Furthermore, a relatively high number of patients initially consented but did not complete the questionnaires, raising concerns about barriers to participation and potential response bias. Investigating reasons for noncompletion would be necessary for improving retention in future studies.

The findings reflect short-term outcomes measured 1 day after hospital visits, which may not capture the longer-term effects of podcast interventions on outcomes. Additionally, the reliance on self-reported data introduces the risk of response bias. As this study followed a pragmatic pre-post implementation design, analyses were conducted according to group allocation rather than confirmed exposure to the podcast, consistent with an intention-to-treat approach. Engagement with the podcast was assessed through self-report, and not all patients in the intervention group may have listened to the content. This incomplete exposure may have attenuated the observed effects and could partly explain the modest effect sizes observed. While the exclusion of patients with severe mental illness, cognitive disabilities, or terminal illness was necessary to ensure internal validity, it limits the applicability of results to more vulnerable patient groups.

Finally, variations in how patients engage with and perceive podcast content may have influenced the intervention’s overall effectiveness, suggesting that personal preferences and digital literacy could contribute to patient benefit.

### Future Research

Future research should focus on randomized controlled trials to confirm the effectiveness of podcasts in enhancing SDM, reducing anxiety, and improving patient satisfaction. Studies with longer follow-up periods are also needed to assess the long-term impact of podcasts over time. Comparative effectiveness studies are also needed to evaluate how podcasts perform relative to other formats of patient information, such as written leaflets or videos. These studies will be essential for establishing a robust evidence base to guide the integration of podcasts as a supplement to traditional written patient information.

### Conclusion

This quasi-experimental study suggests that informational podcasts, when used as a supplement to traditional written information, may offer modest support for patient engagement in SDM and improve satisfaction with previsit information, particularly among patients with low health literacy. Effects on previsit anxiety were inconclusive, likely reflecting the multifactorial nature of anxiety and variations in patients’ engagement with digital content. Overall, the impact of the podcasts was modest, indicating that they function primarily as a supplementary tool to traditional written information, rather than as a standalone intervention, and underscoring the importance of identifying patient subgroups who may derive the greatest benefit.

## Abbreviations

COPD: chronic obstructive pulmonary disease
LUP: Landsdækkende Undersøgelse af Patientoplevelser
SDM: shared decision-making
SDM-Q-9: 9-item Shared Decision-Making Questionnaire
STAI-S: State-Trait Anxiety Inventory-State
